# Anatomy of the lobula complex in the brain of the praying mantis compared to the lobula complexes of the locust and cockroach

**DOI:** 10.1002/cne.24208

**Published:** 2017-04-21

**Authors:** Ronny Rosner, Joss von Hadeln, Tobias Salden, Uwe Homberg

**Affiliations:** ^1^ Institute of Neuroscience, Henry Wellcome Building for Neuroecology, Newcastle UniversityFramlington PlaceNewcastle Upon TyneNE2 4HHUnited Kingdom; ^2^ Department of Biology, Animal PhysiologyPhilipps‐University35032MarburgGermany

**Keywords:** cockroach, insect visual system, lobula complex, locust, praying mantis, RRID: AB_2336990, RRID: AB_2315425, RRID: AB_2338713, RRID: AB_2338006, RRID: AB_2313575, RRID: AB_2337244, RRID: AB_2314457, RRID: AB_261363, RRID: AB_2315056, RRID: nif‐0000‐00262

## Abstract

The praying mantis is an insect which relies on vision for capturing prey, avoiding being eaten and for spatial orientation. It is well known for its ability to use stereopsis for estimating the distance of objects. The neuronal substrate mediating visually driven behaviors, however, is not very well investigated. To provide a basis for future functional studies, we analyzed the anatomical organization of visual neuropils in the brain of the praying mantis *Hierodula membranacea* and provide supporting evidence from a second species, *Rhombodera basalis*, with particular focus on the lobula complex (LOX). Neuropils were three‐dimensionally reconstructed from synapsin‐immunostained whole mount brains. The neuropil organization and the pattern of γ‐aminobutyric acid immunostaining of the medulla and LOX were compared between the praying mantis and two related polyneopteran species, the Madeira cockroach and the desert locust. The investigated visual neuropils of the praying mantis are highly structured. Unlike in most insects the LOX of the praying mantis consists of five nested neuropils with at least one neuropil not present in the cockroach or locust. Overall, the mantis LOX is more similar to the LOX of the locust than the more closely related cockroach suggesting that the sensory ecology plays a stronger role than the phylogenetic distance of the three species in structuring this center of visual information processing.

**Abbreviations**
ALO: anterior lobe of the lobula complex
ALO‐D: dorsal compartment of the anterior lobe
ALO‐V: ventral compartment of the anterior lobe
CB: central body
CBL: lower division of the central body
CBU: upper division of the central body
DLO: dorsal lobe of the lobula complex
ILO: inner lobe of the lobula complex
LOX: lobula complex
OLO: outer lobe of the lobula complex
PB: protocerebral bridge
SLO: stalk lobe of the lobula complex

## 1 Introduction

The praying mantis is an ambush predator relying on vision to capture its prey, to orient in space and to avoid becoming the prey of a bird or another predator itself. It has received considerable attention in behavioral studies investigating mating behavior, prey recognition, distance perception, prey capture, and defensive behavior (Prete, 1999). Praying mantises are the only insect species known to use stereopsis for estimating distances, that is, they use binocular disparity for distance perception (Nityananda et al., 2016; Rossel,^1983^). When hunting for prey, a mantis can sit motionless for days hidden in vegetation. When a prey insect approaches, the mantis tracks the insect by head movements, repositions its forelegs, and performs a fast strike with its forelegs only when the object is within reaching distance and in its binocular visual field (Kral & Prete, 1999). In contrast to a fairly good understanding of the behavioral aspects of prey recognition and prey catching, relatively little is known about the neuronal machinery underlying stereoscopic distance estimation and prey capture.

The compound eyes are specialized for stereopsis in several ways. The eyes are large, forward‐directed and consist of about 9,000 ommatidia (Kral & Prete, 1999). As shown in *Tenodera australasiae*, the total field of view of both eyes covers all directions except a small spot in the neck region (Rossel, 1979). Frontally, both eyes show a considerable binocular overlap of about 70° (Rossel, 1979, 1986). The frontal flattening of the eyes results in a reduction of the inter‐ommatidial angle in the frontal eye region to less than 1° and has thus been considered a visual fovea (Rossel, 1979).

How visual signals from the two eyes are integrated in the brain to enable stereopsis, however, is unknown. Like in other insect species, visual signals are processed in three distinct neuropils in the optic lobe: the distal lamina, the medulla, and the proximal lobula complex (LOX; Leitinger, Pabst, & Kral, 1999; Strausfeld, 2012). The lamina and the medulla are layered neuropils with retinotopic organization, similar to their arrangement in other insect species. In contrast, the mantis LOX is compartmentalized into several distinct substructures, unlike its organization in many other insect taxa. Whereas the LOX consists of a single neuropil in bees, two distinct neuropils, termed lobula and lobula plate, are present in flies, butterflies, and beetles, while multiple nested neuropils have been distinguished in locusts and grasshoppers (Ito et al., 2014). The internal organization of the LOX in a praying mantis was first described by Cloarec (1968). She distinguished four distinct subunits in *Mantis religiosa*. Likewise, Leitinger et al. (1999) described four subunits in *Tenodera sinensis*, while Strausfeld (2012) recognized three LOX subdivisions with retinotopic organization.

Work in flies showed that the lobula and lobula plate serve distinct roles. The lobula plate is involved in global motion vision with topographic organization of cardinal motion directions represented in four layers (Borst, Haag, & Reiff, 2010; Borst & Helmstaedter, 2014) whereas the lobula serves a role for small target detection, visual fixation, and figure‐ground discrimination (Aptekar, Keleş, Lu, Zolotova, & Frye, 2015; Lin et al., 2016; Nordström & O'Carroll, 2006; Trischler, Boeddeker, & Egelhaaf, 2007). In contrast, only little information is available on parallel processing of visual information in the LOX subunits in locusts (e.g., regarding polarization vision, Homberg et al., 2011) and no data exist for praying mantises. Although a variety of motion‐sensitive neurons were characterized in the LOX of *M. religiosa* (Berger, 1985) and *Tenodera aridifolia* (Yamawaki & Toh, 2003), the authors did not identify the specific arborization domains of these neurons in the LOX. To provide a basis for functional studies on stereoscopic vision in praying mantises, we have analyzed the anatomical organization of centers for visual processing in two mantis species, focusing on the LOX because of its enigmatic structure and key position in visual information processing. In addition, we compared the organization of LOX subunits with those of two related insect species, the Madeira cockroach and desert locust, through three‐dimensional neuropil reconstructions and γ‐aminobutyric acid (GABA) immunostaining.

## 2 Materials and methods

### 2.1 Animals

Female adult praying mantises (*Hierodula membranacea*, *Rhombodera basalis*) were obtained from colonies of Katharina Wüst (M&m Wüst, Mühlheim Germany) and from a mantis stock at Newcastle University. The animals were kept at temperatures between 22°C and 30°C. Desert locusts (*Schistocerca gregaria*) of both sexes and male cockroaches (*Rhyparobia maderae*) were obtained from crowded colonies at Philipps‐University of Marburg. The animals were kept at a light–dark cycle of 12:12 hr and 50% atmospheric humidity. Locusts were kept at 28°C and cockroaches at 20–26°C.

### 2.2 Immunolabeling of wholemounts

Animals were cold‐anesthetized to 4°C prior to dissection. For immunolabeling of synapse‐dense areas brains were prefixed for 10–60 min in 4% formaldehyde (FA) in 0.1 M phosphate‐buffered saline (PBS; pH 7.4) at room temperature inside the head capsule to minimize tissue distortions and dislocation of the optic lobes (OLs). After dissecting the brains in PBS, they were fixed overnight in 4% FA/PBS at 4°C. After rinsing in 0.1 M PBS containing 0.3% (locust, cockroach) or 5% (mantis) Triton X‐100 (PBT; pH 7.4), the ganglionic sheath of the brain was made permeable by treatment with 1 mg/ml collagenase‐dispase (in 0.05 TRIS‐HCl, pH 7.6) for 1 hr. Following another washing step, all brains were preincubated over night with 5% normal goat serum (NGS; RRID: AB_2336990) in PBT at 4°C. To visualize neuropils, brains were incubated for 5–6 days at 4°C with a monoclonal antibody against the synaptic protein synapsin (SYNORF1, RRID: AB_2315425, kindly provided by Dr. E. Buchner, Würzburg, Germany) diluted at 1:50 in 0.1 M PBT, 1% NGS. The anti‐synapsin antibody is a monoclonal antibody raised in mouse against fusion proteins consisting of glutathione‐s‐transferase and the *Drosophila* SYN1 protein (Klagges et al., 1996). It labels synaptic neuropils as shown in *Drosophila* (Klagges et al., 1996), honeybees (Brandt et al., 2005), and locusts (Kurylas, Rohlfing, Krofczik, Jenett, & Homberg, 2008; Leitinger, Pabst, Rind, & Simmons, 2004). The brains were then washed 3 × 10 min with PB. Secondary antibody, Cy5‐conjugated goat anti mouse or Cy3‐conjugated goat anti mouse (Cy5: RRID: AB_2338713, Cy3: RRID: AB_2338006; Jackson ImmunoResearch, Westgrove, PA), was used at a dilution of 1:300 in PBT, 1% NGS, and applied to the brains for up to 3 days. After rinsing again in PBT (2 × 20 min) and PBS (3 × 20 min), all brains were dehydrated in an ethanol series (25, 50, 70, 90, 95, and 100%, 15 min each), prepared for preclearing in a solution of 50% ethanol and 50% methyl salicylate, and cleared with pure methyl salicylate (Merck, Darmstadt, Germany) until transparent (at least 20 min). Finally, the brains were mounted in Permount (Fisher Scientific, Pittsburgh, PA) between two glass cover slips (24 × 60 mm), which were separated by spacing rings to avoid compression.

### 2.3 Single cell labeling

In four animals, neurons were injected with Neurobiotin (RRID: AB_2313575) by means of intracellular micropipettes drawn on a micropipette puller (P‐97, Sutter Instrument, Novato CA). The tip of the recording electrode was filled with 4% Neurobiotin in 1 mol/l KCl, and Neurobiotin was passed into single neurons through the tip of the electrodes by positive current (0.2–1 nA for several min). The brain was dissected and fixed overnight. For visualization of the neurons the samples were incubated with streptavidin‐Cy3 (1:1,000; RRID: AB_2337244) for 3 days and afterwards dehydrated, cleared and mounted as described above.

### 2.4 GABA immunostaining

Animals were cold anesthetized for 1 hr before their brains were dissected in PBS. Brains were immersed for 2 hr at room temperature in GPA fixative (25% glutardialdehyde, 74% saturated picric acid, 1% acetic acid). After fixation, they were washed with 0.1 M sodium phosphate buffer (NaPi). The brains were embedded in a gelatin/albumin mixture and then post‐fixed overnight at 4°C in 8% PFA. Post‐fixed tissues were sliced frontally with a vibrating blade microtome (Leica, VT 1200S, Bensheim, Germany) at 30 µm thickness. The sections were preincubated for 1 hr in 8% NGS in SST (saline substituted TRIS‐buffer containing 1% Triton X‐100) at room temperature, followed by incubation in antiserum raised in rabbit against GABA. The anti‐GABA antiserum (RRID: AB_2314457, kindly provided by Dr. T.G. Kingan, University of Arizona, Tucson, AZ) was diluted 1:6,000–:10,000 in SST with 2% NGS and applied to the sections for 1 hr at 37°C followed by 2 days at 4°C. After incubation, the sections were washed 3 × 10 min in SST. The second antibody (goat anti‐rabbit IgG; RRID: AB_261363) was applied at a concentration of 1:40 in SST containing 2% NGS for 2 hr at room temperature. After washing for 3 × 10 min in SST, the sections were incubated in rabbit peroxidase‐anti peroxidase (rabbit‐PAP, RRID: AB_2315056) at a concentration of 1:300 in SST containing 1% NGS for 1 hr at room temperature. After incubation, the sections were washed again for 3 × 10 min in SST. The sections were stained by incubation in a solution of 3,3′‐diaminobenzidine tetrahydrochloride (1:30 in NaPi with 0.5% H_2_O_2_) for 5 to 45 min. When a dark brown reaction product had developed, the sections were washed for 3 × 10 min in NaPi and were, finally, mounted on microscope slides coated with chrome alum gelatin. After drying, the sections were dehydrated in an ethanol series (25, 50, 70, 90, 95, and 100%, 15 min each), cleared in xylenes and embedded in Entellan under cover slips.

### 2.5 Specificity of the GABA antiserum

The specificity of the anti‐GABA antiserum (RRID: AB_2314457) has been characterized in the sphinx moth *Manduca sexta* (Hoskins, Homberg, Kingan, Christensen, & Hildebrand, 1986) and the desert locust *S. gregaria* (Homberg, Vitzthum, Müller, & Binkle, 1999). Liquid phase preadsorption of the diluted antiserum with 60 nM GABA‐BSA (bovine serum albumin) conjugate or 24 nM GABA‐KLH (keyhole limpet hemocyanin) conjugate abolished immunostaining on paraffin sections of *M. sexta*, and preadsorption with 15 nM GABA‐BSA conjugate abolished immunostaining on gelatin‐embedded sections of *S. gregaria*. Here, preadsorption of the diluted (1:8,000) anti‐GABA antiserum with 50 µM of GABA‐glutaraldehyde complex, prepared as described by Ottersen, Storm‐Mathisen, Madsen, Skumlien, and Strømhaug (1986) abolished all immunostaining on gelatin‐embedded sections from *H. membranacea*.

### 2.6 Image acquisition and processing

Whole mounts were scanned with a confocal laser scanning microscope (CLSM, TCS SP5, Leica Microsystems, Wetzlar, Germany) with a 10 × oil immersion objective lens. Most image stacks were generated with a scan velocity of 200 Hz and a format of 1024 × 1024. A line average of 2 was used. The Cy3 and background signal was detected with a DPSS (561 nm) laser while Cy5‐fluorescence was detected with a HeNe (633 nm) laser. The data stacks were processed with Amira 5.33 (Advanced 3D Visualization and Volume Modeling, RRID: nif‐0000‐00262). Images from the peroxidase‐labeled preparations were captured using a compound microscope (Zeiss Axioskop) equipped with a digital camera (ProgRes C12plus, Jenoptik, Jena, Germany). The size, contrast, and brightness of the images were adjusted using Photoshop CS5 (Adobe Systems, Ireland). The composition of the figures and the lettering was done using Illustrator CS5 (Adobe Systems, Ireland).

### 2.7 Three‐dimensional reconstructions

Three‐dimensional reconstructions of neuropils were performed manually with Amira 5.33 based on anti‐synapsin labeling and in some preparations background staining. Characteristic vertices of the neuropils were marked on different levels of the image stacks for subsequent computation of the structures. Polygonal surfaces were created with the module SurfaceGen. Neuronal reconstructions were done with the SkeletonTree tool within Amira 5.33 (Evers, Schmitt, Sibila, & Duch, 2005; Schmitt, Evers, Duch, Scholz, & Obermayer, 2004).

## 3 Results

### 3.1 Synapsin‐stained neuropils in the praying mantis protocerebrum

**Figure 1:**
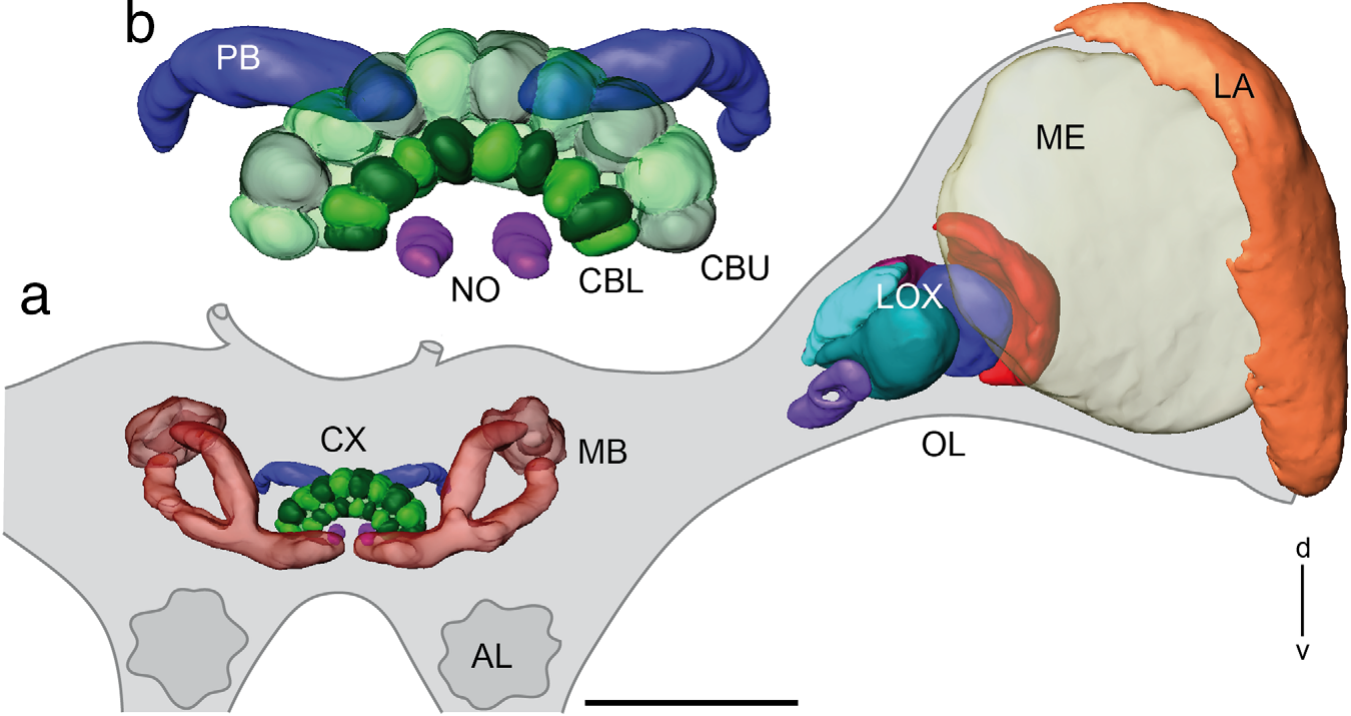
Three‐dimensional reconstructions of principle neuropils in the protocerebrum of the praying mantis *Hierodula membranacea*. (a) Frontal view of reconstructed neuropils of the central brain and the left optic lobe embedded in the outlined mantis brain. (b) Enlarged view of the reconstructed central complex with central body (CB), PB, and paired noduli. AL = antennal lobe; CBL = lower division of the CB; CBU= upper division of the CB; CX = central complex; d = dorsal; LA = lamina; LOX = lobula complex; MB = mushroom body; ME = medulla; NO = noduli; OL = optic lobe; PB = protocerebral bridge; v = ventral. Scale bar = 500 µm

The brain of the praying mantis is characterized by large OLs, slender optic stalks, and a relatively small central brain (Figure 1a). The neuropils of the two OLs contribute most to the volume of the mantis protocerebrum. The central complex is, likewise, relatively large in comparison to the mushroom bodies. It consists of a medially constricted protocerebral bridge (PB), a central body (CB), and a pair of noduli (Figure 1b). The CB is further divided into a lower and an upper division (CBL, CBU) which correspond to the *Drosophila* ellipsoid‐ and fan‐shaped body, respectively. The CBL consists of an anterior and a posterior layer while the CBU appears as a single layer in synapsin labeling. The anterior layer of the CBL and the CBU are composed of 10 cone‐like modules, 5 in each brain hemisphere that possibly correspond to the slices in the CB of other species. The outermost cones are reduced in thickness to about half of the size of the remaining ones. No layers could be identified in the PB and noduli.

The mushroom bodies have a small double cup‐shaped calyx pointing dorso‐posterior, and a slender peduncle, which divides equally into a dorsally projecting vertical lobe and a medial lobe (Figure 1a). An anterior optic tubercle could not be recognized.

**Figure 2:**
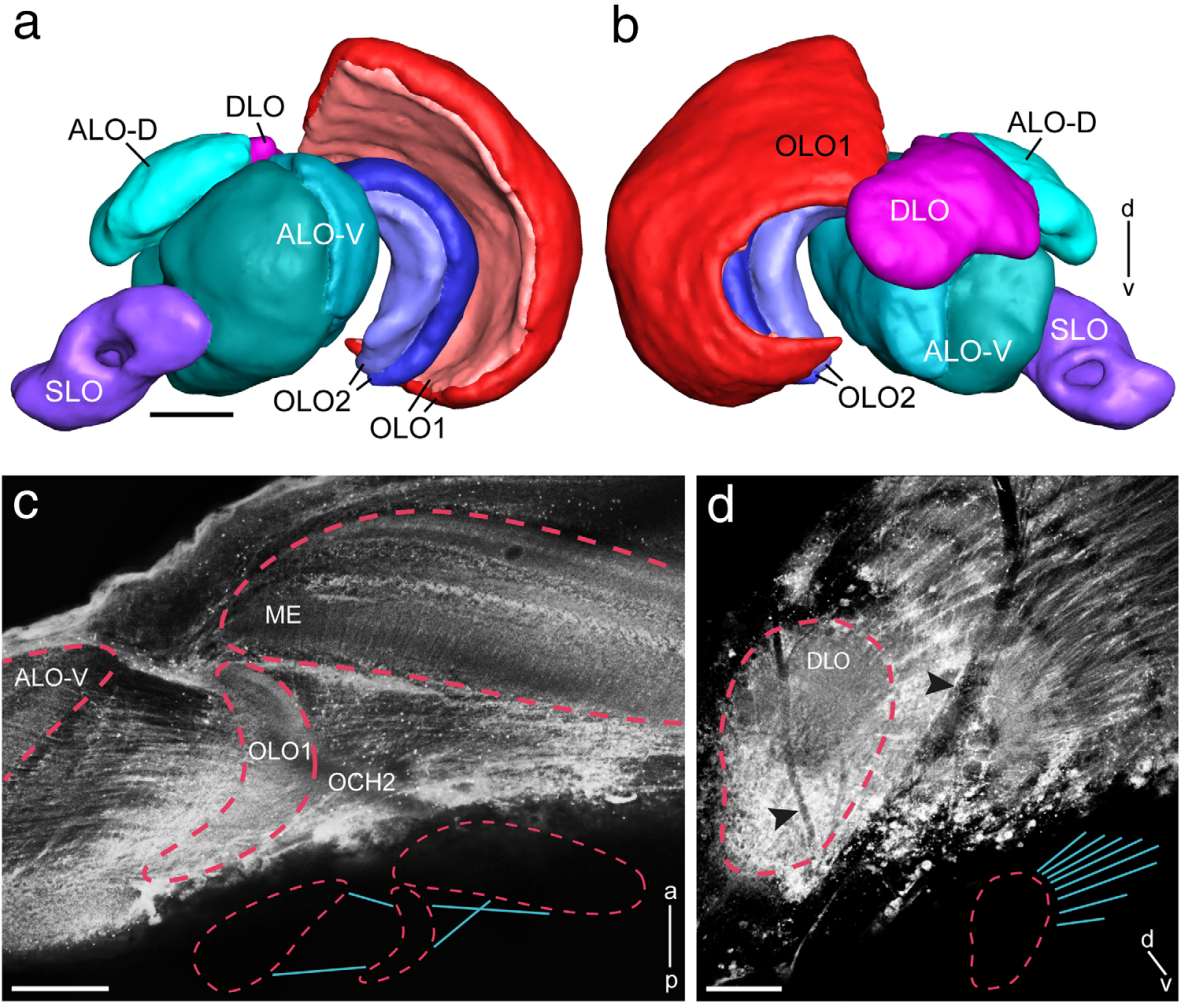
Detailed layout of the lobula complex (LOX) in *Hierodula membranacea*. (a, b) Three‐dimensional reconstruction of LOX neuropils as seen from frontal (a) and posterior (b). (c, d) Projection views of multiple confocal images for visualization of connectivity patterns of key optic lobe neuropils. (c) The OLO1 is connected with the medulla via the second optic chiasm. The ALO receives retinotopic input from the OLO1 via uncrossed fibers. Medulla, OLO1 and ALO are outlined by red dashed lines. Inset shows outline of connectivity pattern between medulla, OLO1 and ALO. (d) The DLO receives retinotopic from the medulla. The DLO is outlined by red dashed line. Black arrowheads point toward trachea present at the surface of the optic lobe. Inset shows outline of connectivity pattern between DLO and medulla. a = anterior; ALO‐D = dorsal subunit of the anterior lobe; ALO‐V = ventral subunit of the anterior lobe; d = dorsal; DLO = dorsal lobe; ME = medulla; OCH2 = second optic chiasm; OLO1 = outer lobe 1; OLO2 = outer lobe 2; p = posterior; SLO = stalk lobe; v = ventral; Scale bars = 100 µm (a–d)

The OL harbors, as in other insect species, from distal to proximal, the lamina, the medulla, and the LOX (Figure 1a). The lamina is a narrow neuropil beneath the compound eye. It is connected via the first optic chiasm (not shown) to the second optic neuropil, the medulla, which is by far the largest optic neuropil. No specific dorsal rim areas could be identified in the lamina or medulla. The mantis LOX consists of 5 lobes (Figures 1a, 2a,b, and 5d). It strongly resembles the LOX of the locust (compare Figure 5d,e). We, therefore, adopted the nomenclature of LOX subunits as introduced by Gouranton (1964) and used by Kurylas et al. (2008) for the locust, which had already been adapted for the mantis by Cloarec (1968). The most distal subunits of the LOX are the two outer lobes OLO1 and OLO2. They correspond to the capsule postérieure and the capsule postéro‐interne of Cloarec (1968) and Lo1 and Lo2 of Leitinger et al. (1999), and show an internal retinotopic organization. The outer lobe 1 (OLO1) is retinotopically connected to the medulla via the second optic chiasm (Fig. 2c). Both OLO1 and OLO2 have two layers, as has the more proximally located ventral compartment of the anterior lobe (ALO‐V; Figures 2a,b, 4b, and 5d). The ALO‐V is segregated from a dorsal subunit (ALO‐D). The ALO receives retinotopic input from the OLO1 (Figure 2c). The dorsal lobe (DLO) lies dorsally and posteriorly from the ALO. It receives retinotopic input from the medulla (Figure 2d). The most proximal neuropil in the OL is the tunnel‐shaped stalk lobe (SLO; Figure 2a,b). It has not been described previously and has no counterpart in the desert locust. We could not identify clear input structures on the basis of background‐, synapsin‐ or GABA stainings. A nerve bundle passes through the tunnel (not shown).

**Figure 3:**
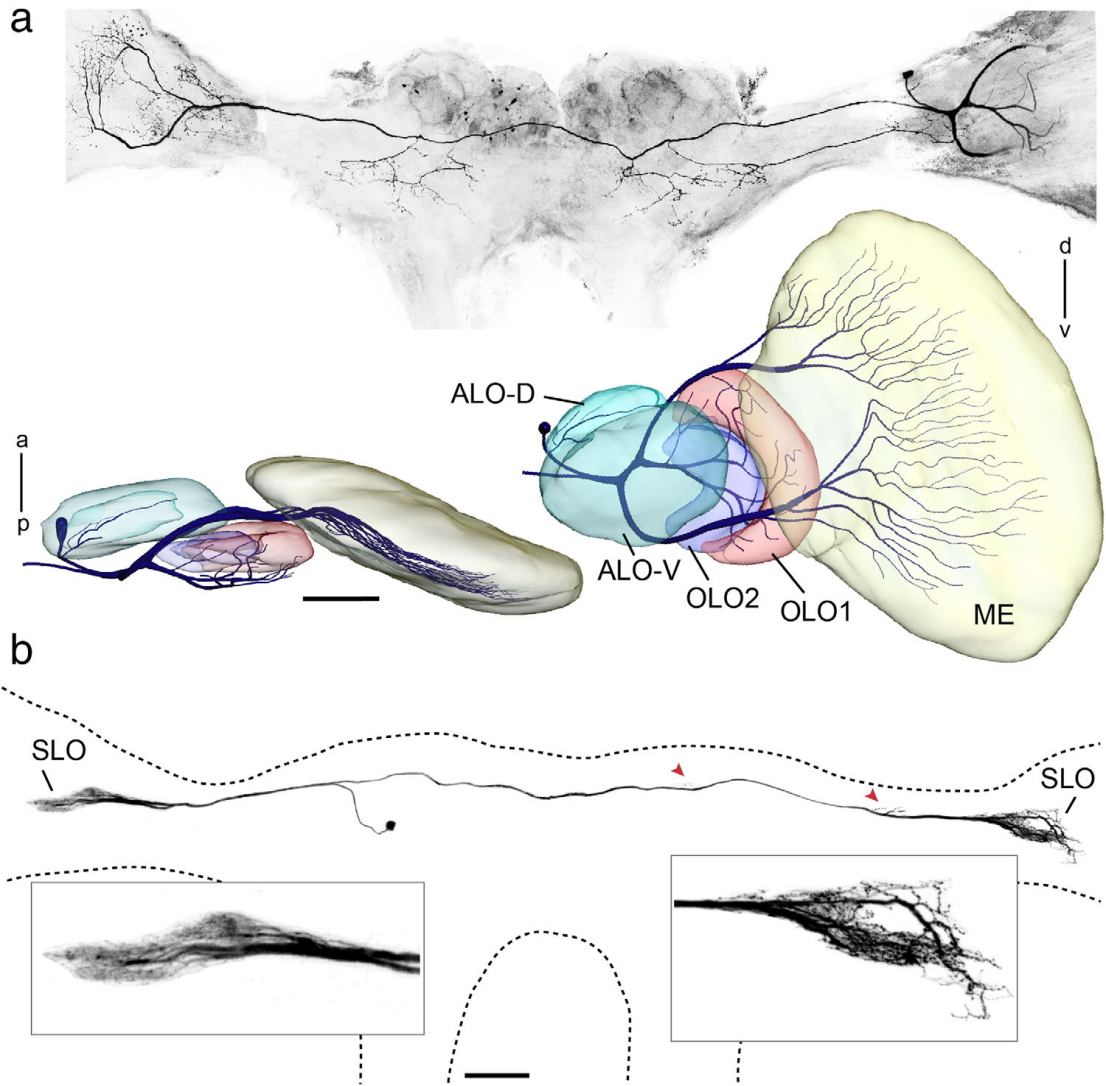
Commissural neurons ramifying in the optic lobes of the praying mantis. (a, b) Neurons stained by injection of Neurobiotin via intracellular micropipettes. (a) Projection view of multiple confocal images of MELOXcom neuron in *Rhombodera basalis*. The medullae are truncated because of poor visibility in projection view and for reasons of space. Below: Frontal and ventral views of three‐dimensional reconstruction of optic lobe neuropils and neuron ramifications in the ipsilateral medulla and LOX. (b) Commissural neuron (SLOcom) ramifying in the stalk lobes (SLO) of both optic lobes and in the dorsal protocerebrum. The sites of arborizations in the dorsal protocerebrum are indicated by red arrowheads. The image of the neuron was generated as projection view from confocal image stack by masking background. Insets show smooth ramifications in the SLO ipsilateral to the cell's soma and beaded endings in the contralateral SLO. a = anterior; ALO‐D = dorsal compartment of the anterior lobe; ALO‐V = ventral compartment of the anterior lobe; d = dorsal; ME = medulla; OLO1 = outer lobe 1; OLO2 = outer lobe 2; p = posterior; v = ventral. Scale bars = 100 µm (a and b)

**Figure 4:**
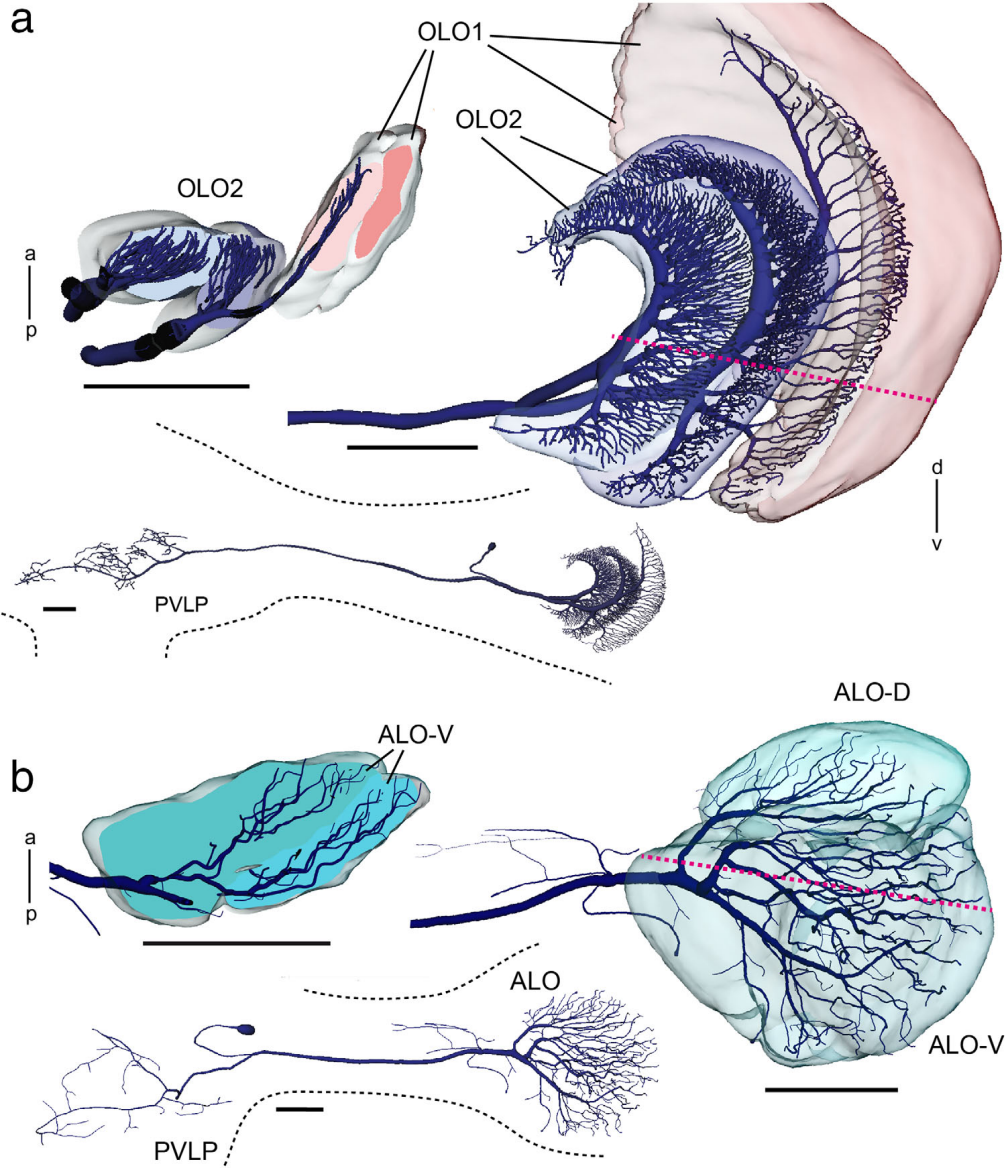
Three‐dimensional reconstructions of tangential neurons ramifying in the LOX of *Hierodula membranacea*. (a, b) Two projection neurons were stained during intracellular recordings. Both neurons have ramifications in the posterior ventrolateral protocerebrum (insets) and the LOX. (a) Projection neuron from the outer lobe, termed TOpro1‐neuron. Extensive dendritic arborizations are confined to the two layers of OLO2 and the most proximal layer of OLO1 as is especially apparent in horizontal profile view. (b) Frontal view and horizontal section showing dendritic tree of projection neuron termed TApro1‐neuron with ramifications in two layers of the ALO‐V and in the ALO‐D. Red dotted lines in (a) and (b) indicate planes of horizontal sections. a = anterior; ALO = anterior lobe; OLO1 = outer lobe 1; OLO2 = outer lobe 2; p = posterior; PVLP = posterior ventrolateral protocerebrum. Scale bars = 200 µm (a, b)

The arborization patterns of individual, Neurobiotin injected neurons support the anatomical segmentation of the LOX which we carried out based on synapsin immunostaining. Four neurons were analyzed through confocal microscopy and reconstructed in three dimensions (Figures 3 and 4). The medulla‐LOX commissural neuron (MELOXcom; Figure 3a) ramifies in both OLs and in the central brain. We only reconstructed the smooth arborizations in the OL ipsilateral to the cell soma which is located in the dorsal rind of the OL. Processes extend into the inner medulla, OLO1 and the ALO‐D. A second commissural neuron, termed SLOcom, connects both SLOs (Figure 3b). It has its soma in the frontolateral protocerebrum. Ramifications are present in both SLOs and in the dorsal frontolateral central brain contralateral to the cell's soma. The arborizations in both OLs are restricted to the tunnel shaped SLO. The OL arborizations ipsilateral to the soma appear to be smooth while the contralateral ramifications are beaded.

In addition, two projection neurons from the LOX were stained and reconstructed (Figure 4). A tangential projection neuron from the OLO, termed TOpro1‐neuron (Figure 4a) has two main branches within the optic lobe. One neurite gives rise to tangential ramifications in the inner layer of OLO2 and the second invades OLO1 and the outer layer of OLO2. The second tangential projection neuron, termed TApro1‐neuron (Figure 4b) ramifies in all sub‐compartments of the ALO and has additional ramifications in the optic stalk. Fine dendritic ramifications of this neuron extend both to the ALO‐D and the ALO‐V (see horizontal section in Figure 4b).

### 3.2 Comparison of the LOX in the praying mantis, locust, and cockroach by means of synapsin staining

**Figure 5:**
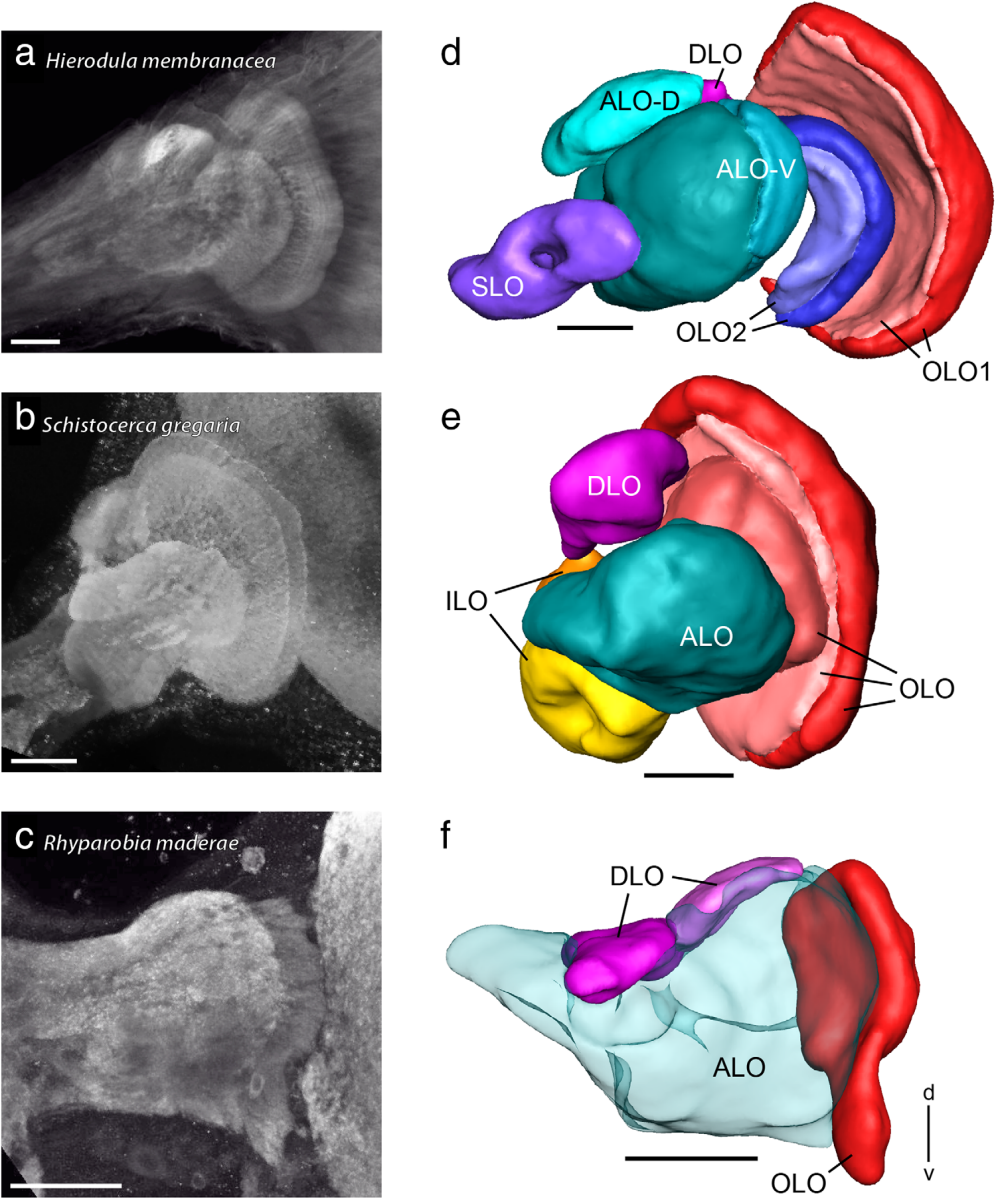
Lobula complexes (LOXs) of the praying mantis *Hierodula membranacea*, the locust *Schistocerca gregaria*, and the cockroach *Rhyparobia maderae*. (a–c) Projection views of synapsin immunostained LOXs of the praying mantis (a), locust (b), and cockroach (c) obtained from confocal image stacks. (d–f) Three‐dimensional reconstructions of the LOXs shown in (a–c). ALO = anterior lobe; ALO/ILO = anterior lobe inner lobe aggregate; d = dorsal; DLO = dorsal lobe; ILO = inner lobe; OLO = outer lobe; OLO1 = outer lobe 1; OLO2 = outer lobe 2; SLO = stalk lobe; v = ventral. Scale bars = 100 µm (a–f)

Based on synapsin immunolabeling, the neuropils of the LOXs of the locust *Schistocerca gregaria* and cockroach *Rhyparobia maderae* were reconstructed three‐dimensionally for comparison with the LOX in the praying mantis (Figure 5). The LOXs of the praying mantis (Figure 5a,d) and locust (Figure 5b,e) are highly compartmentalized while the subdivisions of the LOX in the cockroach (Figure 5c,f) are less well discernible. All three species have in common a distally located, retinotopically organized OLO and a DLO. The cockroach and locust OLOs are undivided neuropils in contrast to the mantis, in which the OLO is segregated into two nested neuropils (OLO1 and OLO2). Synapsin immunostaining revealed two layers each in the mantis OLO1 and OLO2 (Figure 5d), three layers in the locust OLO (Figure 5e), and a single‐layered OLO in the cockroach (Figure 5f). The DLO in the cockroach is divided into two compartments (Figure 5f) while the DLOs of the locust and mantis are not (see Figure 5e for locust and Figure 2b for mantis DLO).

The remainder of the cockroach LOX could not be segmented further, because of its diffuse appearance. It is referred to as anterior lobe (ALO, Figure 5f) based on its location corresponding to the ALO of the mantis (Figure 5d) and locust LOX (Figure 5e) and similarities in the pattern of GABA immunostaining in all three species (see below). The mantis ALO is further segmented into a dorsal (ALO‐D) and a ventral compartment (ALO‐V). In the mantis ALO‐V two layers can be distinguished on the basis of synapsin immunostaining (Figure 5d). The undivided locust ALO harbors four layers that were recognized already by Homberg, Hofer, Pfeiffer, and Gebhardt (2003) but which we did not reconstruct for this study. The locust has a unique LOX module, the inner lobe (ILO) that was found neither in the praying mantis nor in the cockroach as a clearly segregated nested neuropil. The ILO is located posterior of the ALO and ventrally of the DLO (Figure 5e). The praying mantis LOX harbors the above mentioned SLO (Figure 5d) which was present neither in the locust nor in the cockroach LOX.

### 3.3 GABA immunostaining in the medulla

**Figure 6:**
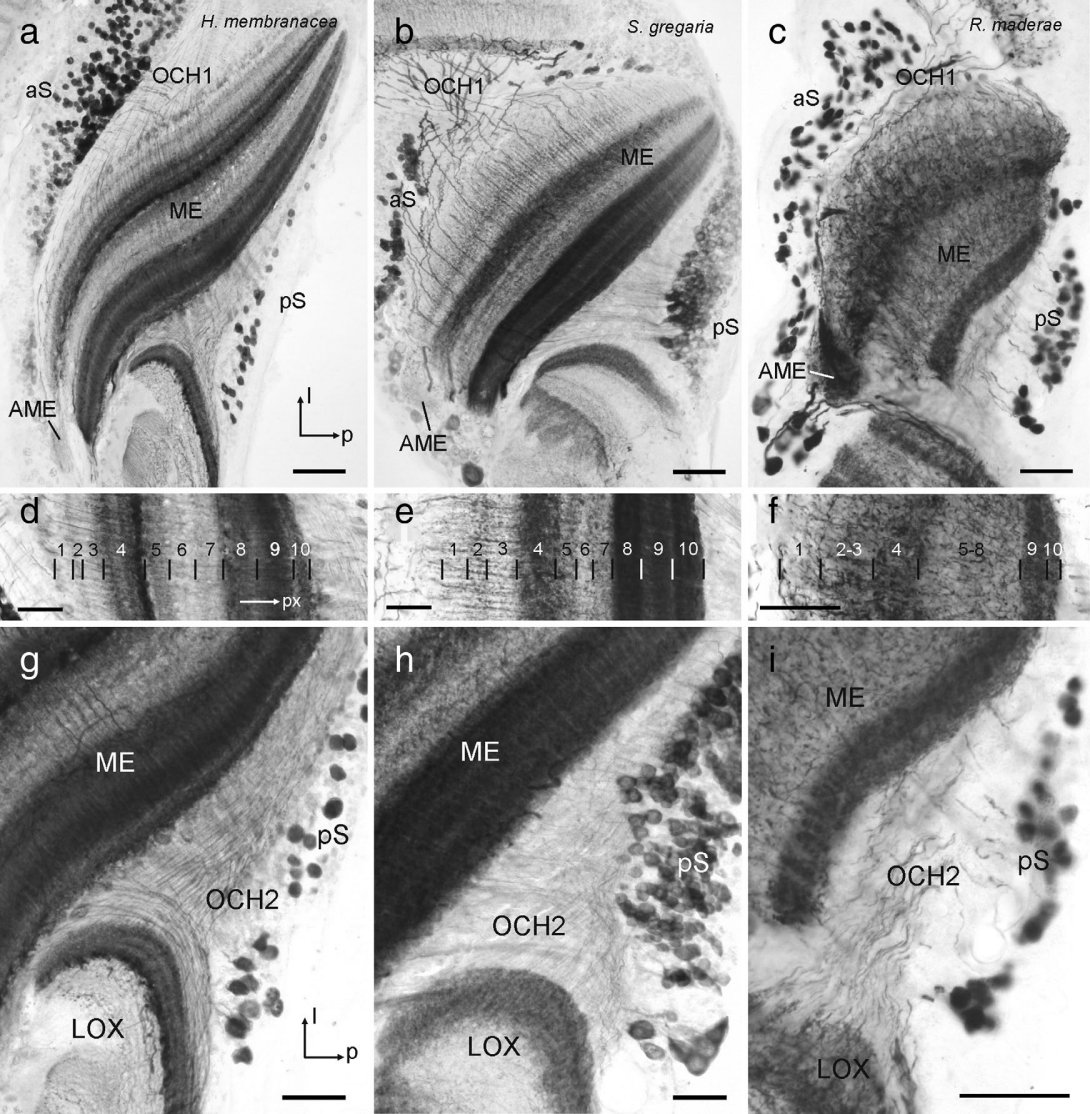
γ‐Aminobutyric acid (GABA) immunostaining in the medulla (ME) and second optic chiasm (OCH2) of the praying mantis *H. membranacea* (a, d, g), the locust *S. gregaria* (b, e, h) and the cockroach *R. maderae* (c, f, i). (a–c) Horizontal sections through the medulla at the level of the accessory medulla (AME). In all three species immunostained somata are scattered in the anterior soma rind (aS) near the first optic chiasm (OCH1) and in the posterior soma rind (pS) adjacent to OCH2. The AME is virtually free of immunostaining in the mantis (a) and locust (b), but shows dense immunostaining in the cockroach (c). l = lateral; p = posterior. (d–f) Horizontal sections through the medulla illustrating the distribution of immunostaining across medulla layers. The ten layers (1–10 from distal to proximal) in the ME of *S. gregaria* conform to previous studies (e.g., Beetz et al., 2015). Layering in *H. membranacea* (d) and *R. maderae* was adjusted to the layering scheme of *S. gregaria* for easier comparison. px = proximal. (g–i) Horizontal sections illustrating fiber trajectories in the second optic chiasm (OCH2) of the three species. Scale bars = 100 µm in (a, b); 50 µm in (c, d–i)

GABA immunostaining was widely distributed throughout the OLs of the three species. Immunostaining in the medulla resulted from numerous scattered cell bodies concentrated in an anterior soma rind adjacent to the first optic chiasm and somata in a posterior soma rind near the second optic chiasm (Figure 6). These neurons gave rise to immunostaining in particular layers of the medulla. In *S. gregaria*, Homberg, Brandl, Clynen, Schoofs, & Veenstra (2004) and Beetz, el Jundi, Heinze, and Homberg (2015) have distinguished 10 layers, numbered 1–10 from distal to proximal. GABA immunostaining was particularly dense in layers 4, 8, 9, and 10, and had a columnar appearance in distal layers 1‐3 (Figure 6b,e). The pattern of immunostaining in *H. membranacea* (Figure 6a,d) was strikingly similar while larger differences exist in *R. maderae* (Figure 6c,f). In *H. membranacea*, like in the locust, the three distalmost layers (1‐3) showed sparse staining with columnar appearance, while layers 4, 8, 9, and 10 were most densely immunolabeled. In contrast to homogeneous staining of layer 4 in the locust, however, the corresponding layer 4 in the mantis showed five fine sublayers, differing in density of GABA staining. In the cockroach *R. maderae*, only 6 layers could be distinguished based on GABA labeling. When adopting a 10‐layer scheme as in the locust, a pattern of labeling with some resemblance to that in the locust and mantis became apparent. Again layer 4 and two proximal layers, 9 and 10 showed most dense GABA immunstaining (Figure 6c,f). A large zone corresponding to layers 5‐8 showed sparse immunostaining without further layering. The three distalmost layers (1–3) were less distinct than in the mantis or locust (Figure 6f). In the locust and the mantis, the accessory medulla was virtually devoid of immunostaining (Figure 6a,b) whereas in the cockroach is was densely supplied by GABA‐immunoreactive processes (Figure 6c). In all three species, immunostained fibers in the second optic chiasm connected the medulla to the LOX (Figure 6g–i). In the locust, these neurites were exceedingly fine (Figure 6h).

### 3.4 GABA immunostaining of LOX neuropils

#### 3.4.1 Hierodula membranacea

**Figure 7:**
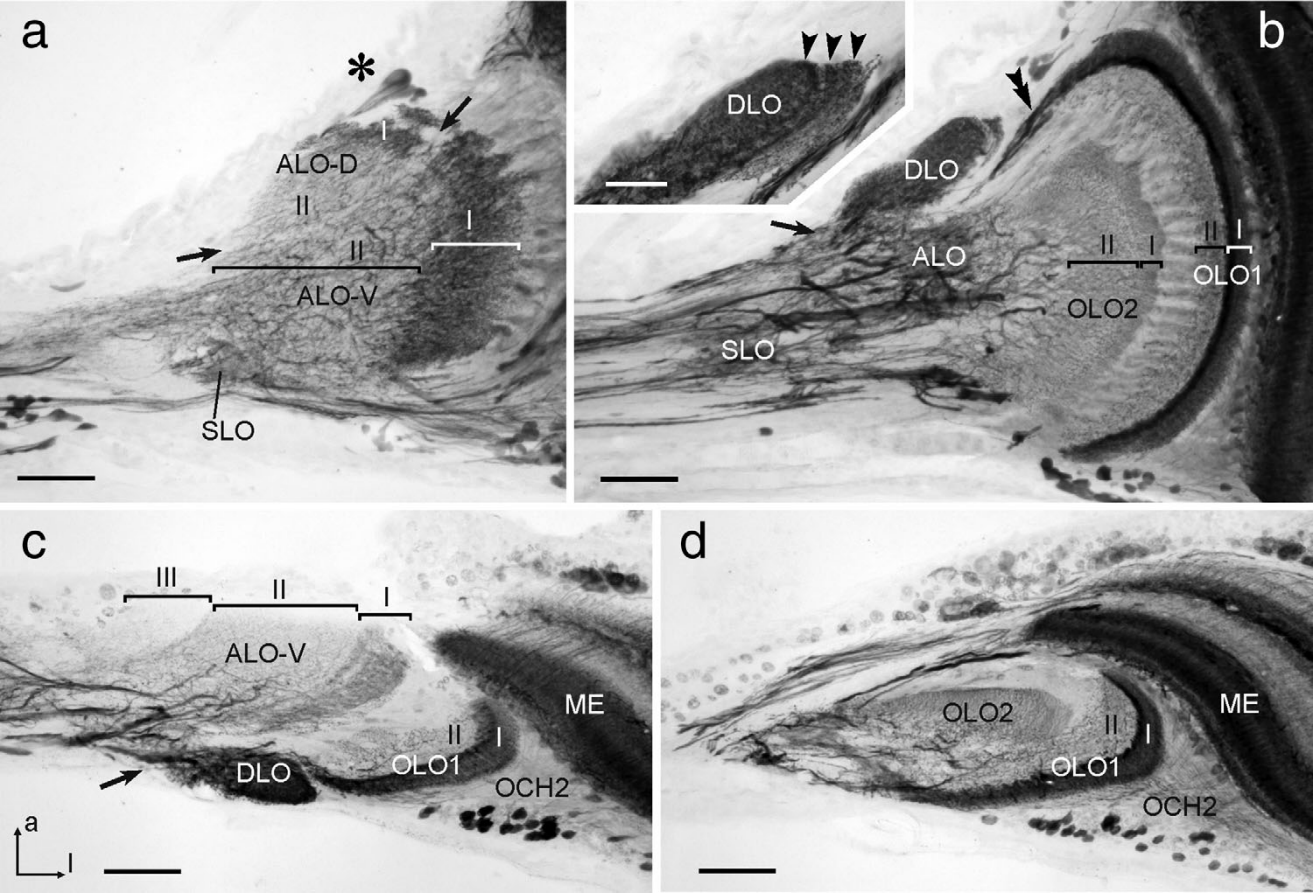
γ‐Aminobutyric acid (GABA) immunostaining in the lobula complex (LOX) of the praying mantis *H. membranacea*. (a, b) Frontal sections at an anterior (a) and an intermediate (b) level through the LOX. (c, d) Horizontal sections at a dorsal (c) and ventral (d) level. Sections illustrate immunostaining in the anterior lobe (ALO‐D, ALO‐V), dorsal lobe (DLO), outer lobe 1 and 2 (OLO1, OLO2), and the stalk lobe (SLO). Inset in (b) illustrates layered organization of the DLO (arrowheads). The anterior lobe can be subdivided into a dorsal and a ventral unit (ALO‐D, ALO‐V, divide indicated by arrows in (a). Based on distinct differences in immunostaining, 2 layers can be distinguished in the ALO‐D, OLO1, and OLO2 (labeled I and II, respectively), and three layers in the ALO‐V (labeled I–III). Asterisk in (a) indicates immunoreactive somata of tangential neurons near the dorsal face of the ALO‐D. Arrows in (b) (except inset) and (c) point to immunostained fibers entering the DLO. Double arrowhead in (a) points to immunoreactive fibers connecting OLO1 with the central brain. OLO1 is, in addition, innervated by columnar neurons from the medulla (ME) with fibers passing through the second optic chiasm (OCH2). a = anterior; l = lateral (applies to c and d). Scale bars = 200 µm; 50 µm in inset of (a)

GABA immunostaining was present in all lobes of the LOX (Figure 7) and supports the organization of the LOX as revealed through synapsin immunostaining. In addition to small immunoreactive cell bodies in the soma rind posterior to the second optic chiasm (Figure 6a,g and 7c), only small numbers of larger cell bodies were immunostained near the dorsal and ventral face of the ALO (asterisk in Figure 7a) and in the optic stalk (not shown). In the OLO1 of the LOX two layers could be distinguished based on different patterns of GABA immunostaining (Figure 7b–d). The distal layer I was densely supplied by GABA‐immunoreactive processes, which originated from neurons entering the outer face of OLO1 from the medulla through the second optic chiasm (Figures 6g and 7d). In addition, processes from tangential neurons, connecting the LOX with the central brain, entered layer I at its dorsal rim (double arrowhead, Figure 7b) and projected in a fan‐shaped manner throughout layer I. Layer II of OLO1, in contrast, was invaded only by one or a few tangential neurons with fine beaded processes extending through layer II. OLO2 was only sparsely stained. It was innervated by finely branching processes from tangential neurons with main fibers in the optic stalk, giving rise to slightly different appearances of immunostaining in layers I and II (Figure 7b). The ALO was innervated by an irregular meshwork of immunostained processes that continued into the optic stalk (Figure 7a). It originated from several fibers in the optic stalk (Figure 7c). As in synapsin immunostaining, a distinction into a smaller dorsal (ALO‐D) and a larger ventral unit (ALO‐V) of the anterior lobe was apparent. However, in contrast to synapsin immunostaining, two instead of a single layer could be distinguished in ALO‐D (Figure 7a) and three instead of two layers in ALO‐V (Figure 7c). Immunostaining was densest in the distal layer I, less dense in layer II and least dense in layer III (Figure 7c). The DLO was innervated from its proximal and ventral edge by immunostained fibers in the optic stalk, which were possibly side branches of neurites that continued into the OLO1 (Figure 7b,c). Like layer I of OLO1, the DLO was densely innervated by beaded processes (Figure 7b,c) that revealed a layered internal organization (Figure 7b, inset). Finally, the SLO was recognized by irregular innervation, apparently largely from side branches of tangential neurons targeting other subunits of the LOX (Figure 7a,b).

#### 3.4.2 *Schistocerca gregaria*

**Figure 8:**
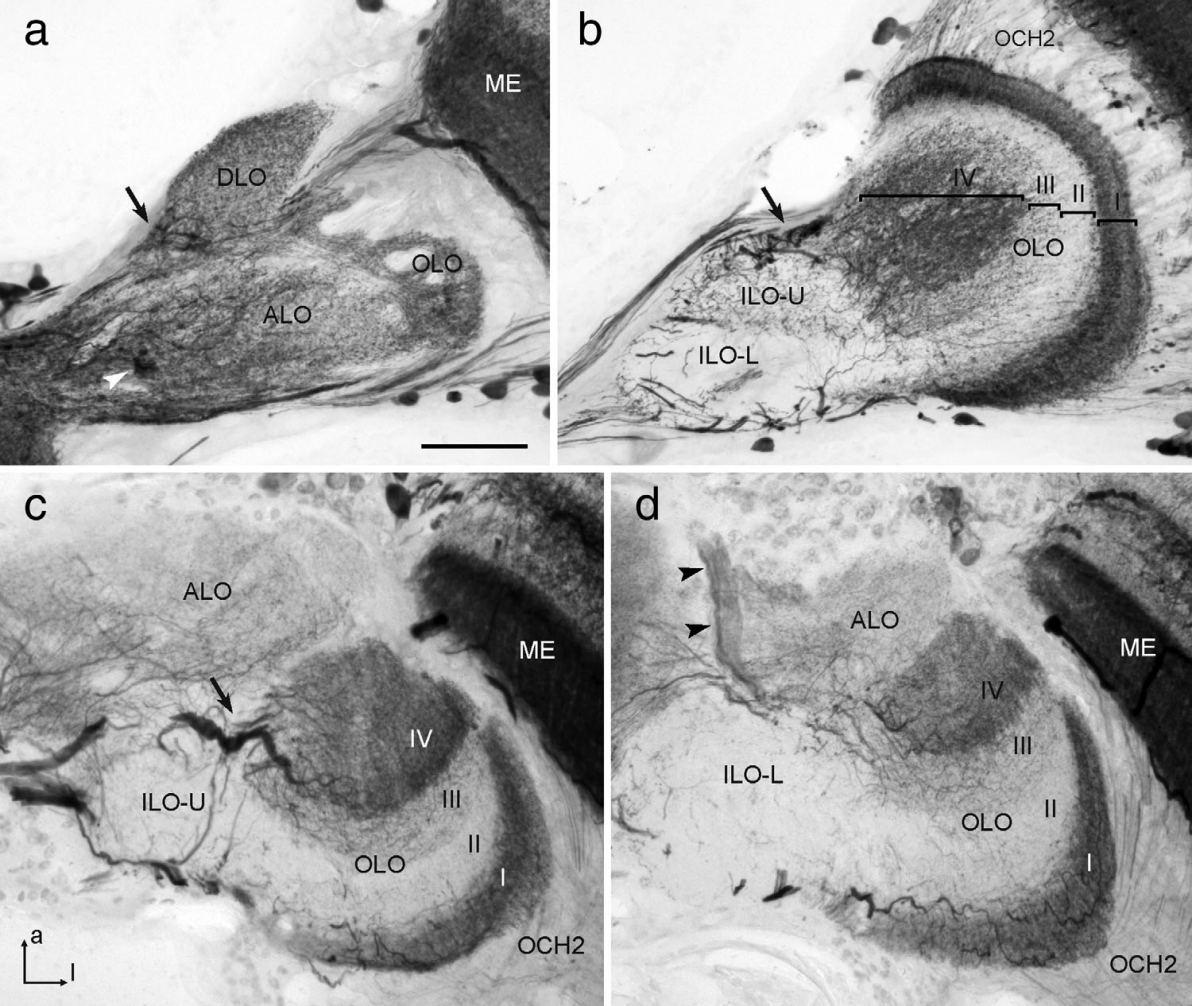
γ‐Aminobutyric acid (GABA) immunostaining in the lobula complex (LOX) of the locust *S. gregaria*. (a, b) Frontal sections at an anterior (a) and a more posterior (b) level; (c, d) horizontal sections at a dorsal (c) and ventral (d) level of the LOX. Distinct immunostaining is present in the anterior lobe (ALO), dorsal lobe (DLO), inner lobe (ILO‐U, ILO‐L), and outer lobe (OLO) of the LOX. In the OLO, four major layers (labeled I–IV) can be distinguished based on different density of GABA immunostaining. The ILO can be further subdivided into an upper half (ILO‐U) with sparse varicose immunostained processes and a lower half (ILO‐L) which is nearly devoid of immunostaining. Arrows in (a, b, and c) point to immunostained fibers entering the DLO and layer IV of the OLO. Arrowheads in (a) and (d) indicate a prominent fiber bundle connecting the ALO with the central brain via the anterior optic tract. Immmunostained fibers in the second optic chiasm (OCH2) connect the medulla (ME) to the OLO. a = anterior; l = lateral (applies to c and d). Scale bar = 100 µm (applies to a–d)

GABA immunostaining in the LOX of the desert locust strongly resembled the staining pattern found in the praying mantis (Figure 8). As in *H. membranacea*, small immunostained cell bodies were scattered in the cell body rind posterior from the second optic chiasm (Figure 6b,h). In addition, small clusters of immunostained somata with primary neurites targeting the LOX were concentrated near the dorsal and ventral edge of ALO (not shown). All neuropils of the LOX showed GABA immunostaining. In contrast to synapsin immunostaining, four instead of only three major layers were distinguished in the OLO based on differences in GABA immunostaining (Figure 8b–d). Its distal layer I showed particularly dense immunostaining, which originated from the projections of columnar neurons entering the outer face of the OLO through the second optic chiasm, and from tangential neurons of the posterior optic tract that invaded layer I at its dorsal edge and gave rise to wide‐field arborizations throughout layer I. Layer II of the OLO was largely free of GABA immunostaining, while the distal layers III and IV showed a fine irregular meshwork of immunostained processes which was slightly more dense in layer IV than in layer III (Figure 8b). The ALO was diffusely innervated by arborizations from a fiber bundle in the anterior optic tract (Figure 8a,d) that bypassed the anterior optic tubercle (not shown). The DLO was uniformly innervated by immunostained processes that entered the unit at its proximal edge and seemed to have side branches extending into layer IV of the OLO (arrows in Figure 8a–c).

#### 3.4.3 *Rhyparobia maderae*

**Figure 9:**
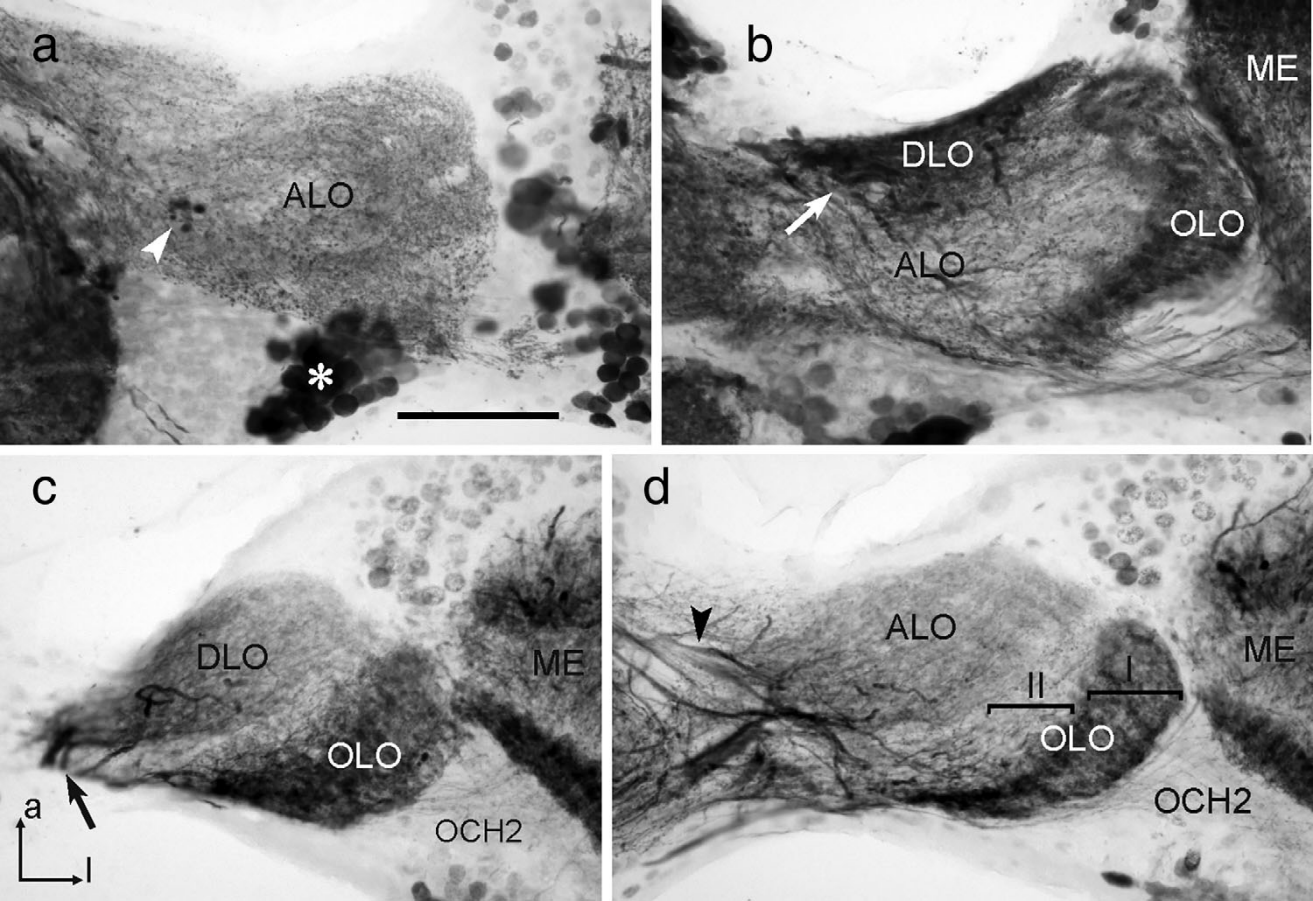
γ‐Aminobutyric acid (GABA) immunostaining in the lobula complex (LOX) of the cockroach *R. maderae*. (a, b) Frontal sections at an anterior (a) and a more posterior (b) level; (c, d) horizontal sections at a dorsal (c) and ventral (d) level of the LOX. Based on distinct GABA immunostaining, an anterior/inner lobe (ALO/ILO), a dorsal lobe (DLO), and an outer lobe (OLO) can be distinguished. In the OLO, a strongly stained distal layer (I) and a sparsely innervated second proximal layer (II) can be distinguished (d). Arrows in (b) and (c) point to immunostained fibers entering the DLO. Arrowheads in (a) and (d) point to a bundle of immunoreactive fibers connecting the central brain and ALO via the anterior optic tract. Asterisk in (a) points to cell bodies whose primary neurites project to the central brain. Immunostained fibers in the second optic chiasm (OCH2) connect the medulla (ME) to the OLO. a = anterior, l = lateral (applies to c and d). Scale bar = 100 µm (applies to a–d)

GABA immunostaining in the LOX of the cockroach reveals features shared with those of the locust and mantis but, as found in synapsin immunostaining, several subunits appeared to be partly fused or reduced. Immunoreactive cell bodies were scattered in the cell body rind facing the second optic chiasm (Figure 6c,i) and sparsely present along the dorsal and anterior face of the LOX. Based on distinct GABA immunoreactivity, an OLO, a DLO, and an ALO could be distinguished (Figure 9). In contrast to the organization revealed through synapsin immunostaining not only one but two layers could be distinguished in the OLO (Figure 9d). The distal layer I was, as in the locust and mantis, strongly stained, while the more proximal layer II was more sparsely innervated by immunostained processes. Like in the mantis and locust, immunostained fibers connected the inner face of the medulla and layer I of the OLO through the second optic chiasm (Figure 6c,i). The ALO was, like in the locust, diffusely innervated by a bundle of immunostained neurites of the anterior optic tract (Figure 9a,d). The DLO showed dense GABA immunostaining originating from fibers of the posterior optic tract that as in the locust seemed to have side branches extending into the ALO (Figure 9b,c). A clear boundary between two DLO subcompartments, as seen in synapsin immunostaining (Figure 5f) was not visible in GABA immunostaining. Compared to the locust and mantis, the DLO was less clearly set apart from the ALO but seemed to be partly fused with the latter.

## 4 Discussion

We have analyzed the neuropil composition and their internal organization of the optic lobe of the mantis *H. membranacea* in comparison to those of the locust *S. gregaria* and the cockroach *R. maderae*. Surprisingly, the layering of the medulla as well as the organization of the LOX is quite similar in the locust and mantis, but shows distinct reductions in the number of discernable medulla layers and LOX subunits in the cockroach. Synapsin immunostaining, single cell analysis, and GABA immunostaining showed that the LOX in the optic lobe of the praying mantis can be partitioned into 5 distinct modules, four of which receive retinotopic input. As judged by relative position, internal organization, and connectivity to other LOX subunits or brain areas, three of these subunits, the OLO, ALO and DLO, have obvious homologs in the desert locust and Madeira cockroach. In addition, one subunit in the locust, the ILO, and one subunit in the praying mantis, the SLO, were not identified in the other species and thus seem to be taxon‐specific. In the central brain, the central complex, a brain area receiving prominent visual input for spatial orientation (Pfeiffer & Homberg, 2014) is large and highly differentiated. In contrast, the anterior optic tubercle, providing massive visual input to the central complex in bees, ants, locusts, and butterflies could not be recognized in the mantis brain.

### 4.1 Medulla layers and LOX subunits in the mantis, locust, and cockroach

The layering of GABA immunostaining in the medulla showed striking similarities between the locust and mantis but appears to be much less differentiated in the cockroach (Figure 6). A particular feature in the mantis was a highly differentiated layer 4 which contrasted against a rather uniformly stained layer 4 in the cockroach and locust. Most dramatic differences were observed in immunostaining of the accessory medulla, which as demonstrated in the cockroach, houses the internal circadian clock of the insect (Stengl & Arendt, 2016). Whether these differences relate to the different activity phases of these insects (cockroach: nocturnal; locust, mantis: diurnal) will have to await further studies.

In all three insects, a distally located, retinotopically organized LOX subunit could be identified which we named outer lobe (OLO). The OLO faces the medulla and receives direct retinotopic input from the medulla via the second optic chiasm (Figure 6g–i). In mantids, the OLO consists of two neuropils (OLO1 and OLO2) with 2 layers each as identified with synapsin‐ as well as GABA immunostainings (Figures 2a and 7). OLO1 and OLO2 correspond to the capsule postérieure and capsule postéro‐interne of Cloarec (1968) and Lo1 and Lo2 of Leitinger et al. (1999), respectively. Two unnamed subunits outlined by Strausfeld (2012) may likewise, correspond to OLO1 and OLO2. In the desert locust, we recognized the OLO as a single subunit, although Gouranton (1964), like Cloarec (1968) in the mantis, recognized two constituents, the capsule postérieure and capsule postéro‐interne. These likely correspond to the distal layers I/II and the proximal layers III/IV of the OLO, distinguished by GABA immunostaining (Figure 8b). Thus, the locust OLO consists of the same number of layers as found in OLO1 and OLO2 of the praying mantis taken together, suggesting that the locust OLO corresponds to the segregated OLO1/OLO2 aggregate in the mantis. However, some differences exist with regard to the relative intensity of GABA immunolabeling in the four OLO layers between locust and mantis. While immunostaining in the most distal layer (layer I of OLO1) is particularly intense in both species, the most proximal layer (layer II of OLO2) is invaded only sparsely by immunoreactive processes in the mantis, but considerably more densely in the locust (Figures 7b and 8b). The cockroach OLO was recognized as a single layer in synapsin staining, but 2 layers were distinguished with GABA immunostaining. As in the mantis and locust, GABA immunostaining is strongest in the most proximal layer (Figure 9d).

The DLO could be identified in all three insect species based upon its unique position and connectivity. Another common feature was its strong GABA immunostaining, originating from fibers entering the DLO at its proximal end. The DLO has at least three layers in the praying mantis, revealed by GABA immunostaining but is differently segregated into two parts in the cockroach illustrated by synapsin staining (Figure 5f). In the locust, the DLO is undivided and neither in the locust nor in the cockroach could we identify a stratification within the DLO.

The most proximal LOX neuropil, the ALO, likewise appears to be homologous in the locust, cockroach and mantis. In all three species, the ALO was partly continuous with the superior lateral protocerebrum. In the praying mantis, it is divided into a dorsal and ventral unit. Leitinger et al. (1999) outlined two anteriorly located LOX neuropils in the praying mantis *T. sinensis* that they referred to as Lo3 and Lo4. They probably correspond to ALO‐D/DLO and ALO‐V in this study and to the vaguely outlined third LOX module in Strausfeld (2012). Cloarec's (1968) capsule antérieure may correspond to the proximal layer of the ALO‐V, while her capsule inféro‐interne may be a conglomerate of the ALO‐D, DLO and distal layer of ALO‐V. We found both the ventral and the dorsal subunit of the ALO having a layered appearance which contrasts with a rather diffuse internal organization in the cockroach. In the locust Homberg et al. (2003) distinguished four ALO layers when comparing FMRFamide‐, tachykinin‐, and leucokinin immunostaining. These layers were not discernable in GABA immunostaining, but most probably correspond to the four layers that were also detectable in synapsin immunostaining of the locust LOX.

Two LOX neuropiles are unique to the praying mantis and the locust, respectively. These are the stalk lobe (SLO) in the mantis (see below) and the ILO in the locust. The ILO flanks the ALO from posterior and is very weakly GABA‐immunoreactive (Figure 8). It exists as a clearly segregated LOX module only in the locust but not in the mantis or cockroach. Except for connections with the OLO and ALO (Elphick, Williams, & O'Shea, 1996; Homberg, 2002; Homberg et al., 2004;), its connectivities to other brain areas and its functional significance have not been resolved. The praying mantis has its own unique LOX neuropile, the proximally located, tunnel shaped SLO. We did not find a corresponding structure in the cockroach nor locust brain. ILO and SLO may subserve specialized visual functions in each taxon. Although vision is the primary modality in both locusts and mantids, they use vision in very different ways. Mantids are visually guided predators and use vision to detect and capture prey, whereas locusts are herbivorous but fly in large groups, where vision is important to avoid collision and to determine the direction of movement relative to the celestial and possibly other spatial cues. The mantis is the only invertebrate known to possess a form of stereoscopic vision, so it is tempting to speculate that the stalk lobe reported here, so far identified only in mantids, might play a role in this ability. Detailed neurophysiological studies would be required to confirm this speculation.

Despite those differences in the neuroanatomical layout of the mantis and locust LOX, the mantis and locust LOX are more similar to each other than either of them is to the cockroach LOX. The same applies to the internal organization in the medulla as revealed by GABA immunostaining (Figure 6). The last common ancestor of cockroach, locust and praying mantis lived about 248 million years ago while the lineage of the more closely related termites, cockroaches and praying mantids separated about 50 million years later (Misof et al., 2014). Why then are the medulla and LOX of mantis and locust much more similar to each other than the LOX of the more closely related species? One possible explanation might be that locusts and mantids share vision as their primary sensory modality while cockroaches rely more strongly on their antennae as mechanosensory and olfactory organs for spatial orientation, food searching and social interaction. The important role of olfaction for cockroaches is reflected in the size of their mushroom bodies, which are huge in comparison to those of *H. membrancea* and locust (Figure 1; Kurylas et al., 2008; Reischig & Stengl, 2002).

### 4.2 Relating LOX modules of mantis, cockroach, and locust to those of the fly

The strongly segregated LOX structure of the praying mantis deviates from the insect optic lobe ground pattern proposed by Strausfeld, 2009, 2012). Strausfeld's pattern includes just two constituents of the LOX, the lobula and lobula plate, as found, for example, in the evolutionary advanced flies. Currently it is unclear whether the highly segregated LOX of the praying mantis is formed by cell types similar to those cells that constitute the LOX of flies. This would mean that the fly LOX would represent a partly fused mantis LOX. Alternatively, some of the mantis and locust LOX constituents might derive from modules located within the central brain in flies and other insect species (see below).

Which structures of the praying mantis LOX could correspond to the two retinotopic substructures of the LOX in flies? The second optic chiasm connecting the medulla and OLO1 in the praying mantis via crossed retinotopic fibers (Figures 2c and 6g) indicates that the OLO might be the equivalent to the fly lobula where similar connections exist. The OLOs in locust, cockroach and mantis are, like the fly lobula, layered when stained with antibodies against GABA (Figures 7, 8, 9; Meyer, Matute, Streit, & Nässel, 1986). The same holds true for the lobula of the honeybee (Meyer et al., 1986). In all five insect taxa, the praying mantis, the locust, the cockroach, the fly and the bee, the most distal of those layers is stained strongly and thus probably contains a high concentration of GABA. Strausfeld refers to part of the cockroach LOX, which might correspond to the DLO, as lobula plate (figure 4.1 in Strausfeld, 2012). The lobula plate in flies receives retinotopic input from the medulla via uncrossed fiber bundles. Retinotopic input from the medulla also exists for the mantis DLO (Figure 2d), but at present, there is no evidence for an internal retinotopic organization of the DLO. The fly lobula plate does not only receive parallel input via the medulla but additionally is provided with uncrossed, retinotopic input from the lobula. We found retinotopic input via uncrossed fibers from the OLO to the ALO in the praying mantis (Figure 2c). Thus, there is the possibility that the fly lobula plate corresponds to the DLO and/or ALO in the praying mantis. However, as for the DLO we were not able to identify retinotopy within the ALO and, therefore, these conclusions remain speculative until corresponding cell types have been identified, for example, via intracellular recordings.

An alternative explanation for the existence of a high number of LOX constituents is that some of them derive from modules located within the central brain in other insect species as was suggested by Elphick et al. (1996) and Strausfeld (2012). These modules could be laterally displaced optic glomeruli. Optic glomeruli receive converging input from ensembles of retinotopic lobula output neurons and are thought to process distinct features of the visual surround (Mu, Ito, Bacon, & Strausfeld, 2012; Strausfeld, Sinakevitch, & Okamura, 2007). Aggregating certain modules involved in processing visual information of particular importance within the OL could improve information processing by shortening travel distances of electrical signals in feed forward and recurrent neuronal connections.

### 4.3 Parallel visual processing in the mantis LOX

The presence, arrangement and connectivity of neuropils in the LOX of the praying mantis suggest that parallel as well as sequential processing of visual signals from the medulla takes place. Behavioral studies show that praying mantises can detect, fixate, and track visual objects by head movements keeping the objects in an acute zone of highest spatial resolution (Prete, 1999; Rossel, 1979) but also perform optomotor responses to large field visual motion (Nityananda et al., 2015). Prey is identified by a combination of visual cues including overall size, contrast to background, location in the visual field and apparent speed (Prete, 1999). Distances are estimated through motion parallax induced by side‐to‐side movements (peering) at larger distances (Poteser & Kral, 1995) and through binocular disparity in the near range (Nityananda et al., 2016; Rossel, 1983, 1986). This illustrates that object‐background discrimination is an essential task of the visual system, as well as specific binocular interactions for distance perception. In contrast, color vision and polarization detection have not been demonstrated and circumstantial evidence indicates that mantises may be monochromatic (Rossel, 1979; Towner & Gaertner, 1994).

How the different LOX subunits contribute to these performances, is not known. Intracellular recordings from LOX interneurons combined with morphological identification of the recorded neuron were achieved by Berger (1985) in *M. religiosa*. He characterized motion‐sensitive neurons responding to a small moving disc, bars, and grating stimuli. Many neurons have a strong preference for small moving objects directly in front of the animal, however some LOX neurons also respond to large field motion as was also found by Yamawaki & Toh (2003). Although the innervated subunits of the LOX were not identified by Berger (1985), all neurons had tangential ramifications in distal areas that might correspond to the OLO or ALO. Many of these neurons had side branches apparently in the DLO or other unidentified proximal regions of the LOX. Axonal projections were in the ipsi‐ or contralateral protocerebrum as shown for the neurons in Figure 4 of this study. The TOpro1‐neuron (Figure 4a) shows high similarity to the nondirectionally motion sensitive L7 cell recorded by Berger (1985). The remaining three cell types of this study were newly identified. Neurons with arborizations in both OLs, as found by Berger (1985) and in this study are promising candidates for being involved in binocular vision.

The current study establishing five distinctly organized subunits in the mantis LOX paves the way for future studies unraveling the distinct functional role of specific LOX subunits in visual tasks.
